# MoMuLV and HIV-1 Nucleocapsid Proteins Have a Common Role in Genomic RNA Packaging but Different in Late Reverse Transcription

**DOI:** 10.1371/journal.pone.0051534

**Published:** 2012-12-07

**Authors:** Célia Chamontin, Bing Yu, Pierre-Jean Racine, Jena-Luc Darlix, Marylène Mougel

**Affiliations:** 1 UMR5236 CNRS, UM1,UM2, CPBS, Montpellier, France; 2 UMR 7213 CNRS, Laboratoire de Biophotonique et Pharmacologie, Faculté de Pharmacie, Illkirch, France; German Primate Center, Germany

## Abstract

Retroviral nucleocapsid proteins harbor nucleic acid chaperoning activities that mostly rely on the N-terminal basic residues and the CCHC zinc finger motif. Such chaperoning is essential for virus replication, notably for genomic RNA selection and packaging in virions, and for reverse transcription of genomic RNA into DNA. Recent data revealed that HIV-1 nucleocapsid restricts reverse transcription during virus assembly – a process called late reverse transcription – suggesting a regulation between RNA packaging and late reverse transcription. Indeed, mutating the HIV-1 nucleocapsid basic residues or the two zinc fingers caused a reduction in RNA incorporated and an increase in newly made viral DNA in the mutant virions. MoMuLV nucleocapsid has an N-terminal basic region similar to HIV-1 nucleocapsid but a unique zinc finger. This prompted us to investigate whether the N-terminal basic residues and the zinc finger of MoMuLV and HIV-1 nucleocapsids play a similar role in genomic RNA packaging and late reverse transcription. To this end, we analyzed the genomic RNA and viral DNA contents of virions produced by cells transfected with MoMuLV molecular clones where the zinc finger was mutated or completely deleted or with a deletion of the N-terminal basic residues of nucleocapsid. All mutant virions showed a strong defect in genomic RNA content indicating that the basic residues and zinc finger are important for genomic RNA packaging. In contrast to HIV-1 nucleocapsid-mutants, the level of viral DNA in mutant MoMuLV virions was only slightly increased. These results confirm that the N-terminal basic residues and zinc finger of MoMuLV nucleocapsid are critical for genomic RNA packaging but, in contrast to HIV-1 nucleocapsid, they most probably do not play a role in the control of late reverse transcription. In addition, these results suggest that virus formation and late reverse transcription proceed according to distinct mechanisms for MuLV and HIV-1.

## Introduction

The retroviral nucleocapsid (NC) corresponds to the C-terminal domain of the Gag polyprotein precursor and found as mature protein upon Gag processing by the viral protease (PR) during virus formation and budding. NC has nucleic acid chaperone activities supported by its basic residues and the zinc finger (ZF) motif (for review, [1], [2]). The basic residues and the ZF domain mediate tight nucleic acid binding in vitro [3], [4]. While NC of betaretroviruses (i.e. Mason-Pfizer Monkey Virus, MPMV), alpharetroviruses (i.e. Rous Sarcoma Virus, RSV) and lentiviruses (i.e. Human Immunodeficiency Virus; HIV) have two ZFs, gammaretroviruses, such as the prototypic Murine Leukemia Virus (MuLV), have only one NC ZF. This unique ZF and the basic residues on its N-terminal side are required for MuLV infectivity [5], [6], [7], [8]. This region plays critical roles in the late phase of MuLV replication since mutating the ZF or deleting the N-terminal basic residues of NC impair packaging of the genomic RNA (gRNA) and virion formation [7], [9], [10], [11], [12], [13]. Dimerization of the gRNA induces a structural RNA switch that exposes conserved UCUG elements that bind NC with high affinity [14], [15], [16]. Such genome recognition by NC promotes the specific packaging of the gRNA in a dimeric form into newly made viral particles [17], [18].

Early after virus infection of target cells, the gRNA is copied by the viral Reverse Transcriptase (RT) to generate the viral DNA in a process called Reverse Transcription (RTion). It is a multistep process initiated from a cellular tRNA annealed to the 5′ end PBS (Primer Binding Site) of the gRNA and subsequently requires two DNA strand transfers to synthesize the complete double-stranded viral DNA flanked by the two long terminal repeats (LTR). Several steps of RTion require nucleic acids remodeling reactions that are chaperoned by NC, notably primer tRNA annealing to the PBS and the two obligatory DNA strand transfers (for review see [19], [20], [21]. Viral DNA synthesis can occur during retrovirus assembly as shown for RSV, MuLV and HIV-1, but at low level ([22], [23], [24]. Recently, mutations in the NC basic residues and ZFs were found to cause extensive RTion in the course of virus assembly in HIV-1 producing cells [25], [26]. Similarly to HBV and foamy viruses, we called this process “late RTion”. Thus, our data further support a role for NC in the control of RTion and its timing throughout the HIV-1 replication cycle [27], [28].

**Figure 1:**
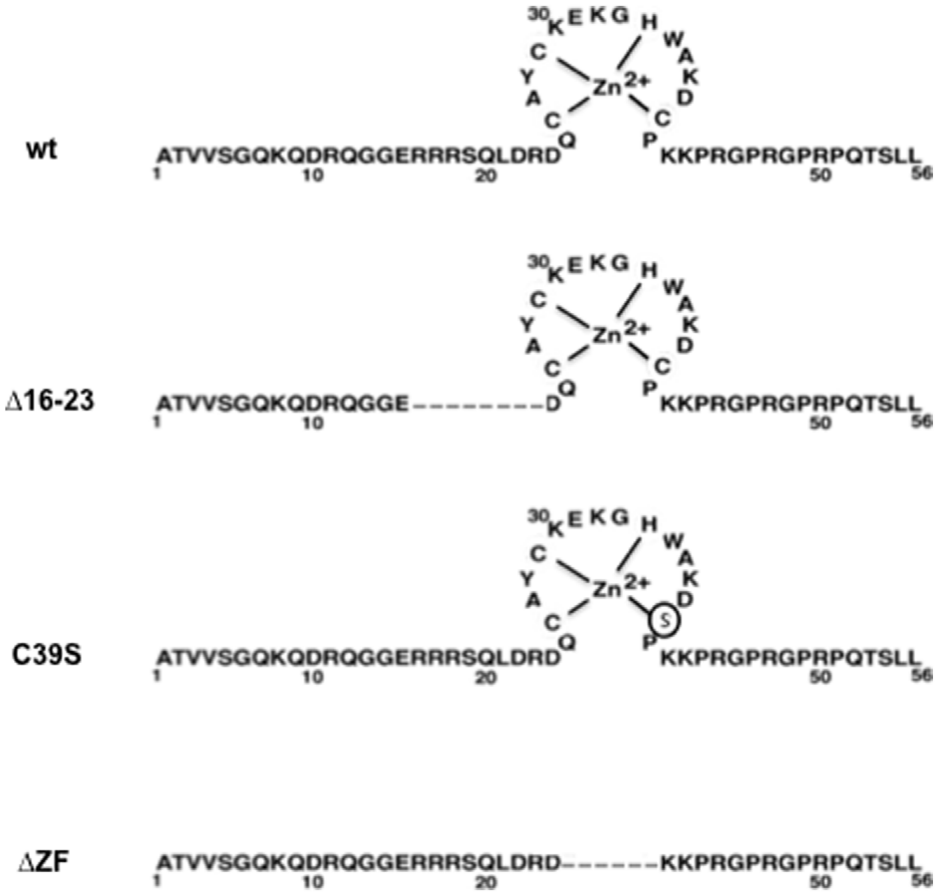
Primary structure of MuLV NC protein and schematic representation of the mutants used here. Numbers indicate amino acid positions. The zinc finger is drawn with the Zn ion coordinated by the CCHC residues. The broken line represents the deleted amino acids.

Yet it is not known whether the involvement of NC in the timing of RTion is specific for HIV-1 or is also valid for other retroviruses, such as alpha- and gammaretroviruses with diverse NCs. Late RTion was maximal when HIV-1 NC contained only the proximal ZF (ZF1) without ZF2 (ΔZF2), indicating that the two ZFs of HIV-1 are not functionally equivalent [26], [29]. However, MuLV has a unique NC ZF that is critical for RTion [30], [31]. To get a better understanding of the role of NC in the late steps of MuLV replication, we asked whether the conserved features of MuLV NC, the basic residues and the unique ZF, are functionally equivalent to those of HIV-1 NC (Fig 1). We revisited by quantitative RT-PCR and PCR analyses the role of MuLV NC in gRNA packaging and we asked whether the basic residues and/or the unique ZF could control RTion during virus assembly. To this end, we studied the impact of mutating or deleting these conserved domains of MuLV NC. Our results show that the basic residues and the unique ZF play a major role in gRNA packaging, and the basic residues (aa16–23, Fig 1) are important determinant for virus release, underlying the similarity between MuLV and HIV NC's. In contrast, MuLV NC, unlike HIV-1 NC, did not influence late RTion since mutating MuLV NC did not cause the accumulation of a high level of viral DNA in mutant virions.

## Materials and Methods

### Plasmids and mutagenesis

The molecular clones pRR88-wt, pRR88-C39S, pRR88-Δ16–23 of Mo-MuLV were provided by A. Rein and previously described in [11], [13], [32]. The MuLV molecular clone PR- contains a deletion of the protease (nt 2421– 2546) as previously described [33]. The pRR88-ΔZF was constructed by substitution of the XhoI-SalI fragment (nt 1560 to 7674 of MuLV) that was previously inserted in a pSP72 vector and mutated with the QuikChange® Lightning Site-Directed Mutagenesis Kit (Stratagene) according to the manufacturer's instructions. The mutated oligonucleotides used were sense-ΔZF (5′-CCCAACTCGATCGCGACAAGAAACCACGAGGA) and antisense-ΔZF (5′-CCTCGTGGTTTCTTGTCGCGGATCGAGTTGGG) to generate construct with deletion of ZF (nt 2127–2174). All constructs were confirmed by sequencing.

The HIV-1 pNL4–3 molecular clone was used to generate the mutant ΔZF2 with harbors a deletion of the second zinc finger motif of the NC. As previouly described [34], this mutant was obtained by site directed mutagenesis using the following olignucleotides: 5′CCTGTCTCTCAGTACCGCCCTTTTTCCTAG3′ and 5′CTTTCATTTGGCATCCTTCC3′, respectively.

### Cell culture, transfection and virus preparation

HEK293T cells were grown in DMEM medium (Dulbecco's modified Eagle's medium) supplemented with glutamine (2 mM), penicillin (100 U/mL), streptomycin (100 µg/mL) and heat-inactivated fetal calf serum (10% v/v) at 37°C. Transfections were performed as previously described [35]. In a standard experiment, 3.5×10^6^ cells were grown in 10 cm dishes. The next day, 8 µg of plasmid DNA were transfected by phosphate calcium precipitation. In all cases, in order to eliminate the plasmid in excess in the medium, the cells were trypsinized 6 hours after transfection, centrifuged and transferred in a new dish. The supernatant was harvested 48h after transfection, centrifuged at 1500 rpm during 10 min and filtered at 0.45 µm. Cells were collected by pipetting with PBS and centrifuged 5 min at 1500 rpm.

### DNA and RNA extractions

Nucleic acids extractions from virions were performed as previously described [26]. Before ultracentrifugation, 400 µl of HIV-1 mutant virions (ΔZF2) obtained as previously described in [26] were systematically added to MuLV supernatants as a tracer to check DNA extraction. However, no tracer was added to the supernatants during the HIV-1 or the HIV-1/MuLV co-expression assays. Then, virions were purified from 15 ml of filtered culture supernatants by centrifugation through a 20% sucrose cushion at 30 000 rpm for 1h 30 at 4°C in an SW32 rotor. Pellets were resuspended in 160 µl of DMEM with 8 U of DNase (RQ1, Promega). One aliquot of virion samples (25µl = 1/6) was saved for virion quantification by Western-Blot analysis as previously in reference [36] and the rest of virions was incubated at 37°C for 45 min to reduce contamination by the transfecting-plasmid DNA. Then, 44 µL of TES 4X (200 mM Tris pH 7.5, 20 mM EDTA, 0.4% SDS) and 20 µg of tRNA carrier were added to the virions before extraction of the nucleic acids by phenol/chloroform and ethanol precipitation.

DNA was extracted from cells with DNAzol (MRC) according to the manufacturer's instructions and as previously described [26]. To avoid any contamination with viral cDNA associated with the particles, cells were extensively washed with cold PBS before DNA extraction. DNA was quantitated by measuring optical absorption at 260 nm.

### RT and qPCR

In vitro reverse transcription was performed as previously described [36] with 1 µg of cellular RNA samples or 1/20 aliquot of virion RNA. Oligo(dT) was used as RTion-primer. A control experiment was systematically performed without RT to look for the absence of DNA contamination. Quantitative PCR assay was achieved with 2.5% of RT reaction or 125 ng of cellular DNA sample and SYBR Green kit (Roche) using the RotorGene (Labgene) systems. The products were amplified by 35 cycles: 95°C for 15s; 60°C for 15s and 72°C for 20s. The following oligonucleotides pairs (0.5 µM) were used: for gRNA and Pol-cDNA, sMLV3350: 5′-TATCGGGCCTCGGCAAGAAAG sense and aMLV3600: 5′-AAACAGAGTCCCCGTTTTGGTG antisense; for ss-cDNA, sensMLV+1: 5′-GCGCCAGTCCTCCGATTGACTGAG sense and aMLV142: 5′-GAAAGACCCCCGCTGACGGGTAGTC antisense; for FL cDNA, sMLV-368 5′-AGAATAGAGAAGTTCAGATC sense and aMLV290 5′- GCTAACTAGTACCGACGCAGGCGC; for SD', sMLV1450: 5′-CTG CTG ACG GGA GAA GAA AAA CA sense and aMLV5620 5′-GCGGACCCACACTGTGTC antisense; for GAPDH, sGAPDH721 5′-GCTCACTGGCATGGCCTTCCGTGT sense and aGAPD931 5′-TGGAGGAGTGGGTGTCGCTGTTGA antisense; for plasmid transfected detection spRR88-784: 5′-CACAGAACTAGTCAGAGACAGCAT sense and aMLV-431: CTTAAGCTAGCTTGCCAAACC antisense, and for specific detection of HIV-1 multi-spliced cDNA (MS cDNA), sHIV5967 =  5′-CTATGGCAGGAAGAAGCGGAG sense and aHIV8527 =  5′-CAAGCGGTGGTAGCTGAAGAG antisense. A standard curve was generated from 50 to 500 000 copies of pRR88-wt plasmid. For each experiment, the DNA purified from virions was checked by a q-PCR assay using the HIV primer pairs (sHIV5967/aHIV8527) specific for the HIV-1 multispliced cDNA forms as previously described [26] to monitor the viral DNA contained in the HIV-1 virions added as tracer. Systematically, cellular GAPDH gene level was determined for standardization of the cellular DNA samples. The background measured from the transfected pRR88 plasmid (spRR88-784/aMLV-431) was deduced to the ss-cDNA.

### Protein analysis

Cells were lysed in presence of Protease inhibitor cocktail (Roche) with the ProteoJet reagent according to the manufacturer's instructions (Fermentas). Total protein concentration was determined by Bradford protein assay using a BSA standard set (Fermentas) and 200 µg of total protein were loaded on 12% SDS-PAGE.

Viral proteins were extracted from 1/6th of virion samples and prepared for gel loading by adding an equal volume of sample buffer (12.5 mM Tris hydrochloride [pH 6.8], 2% SDS, 20% glycerol, 0.25% bromophenol blue, 5% b-mercaptoethanol).

Proteins were analyzed by Western blotting as previously described [36]. Gag proteins were detected with a rat anti-capsid (p30) monoclonal antibody (HyR187; a kind gift from B. Chesebro) used at a 1∶50 dilution as the primary antibody, and a peroxidase-conjugated (HRP) goat anti-rat antibody (1∶2000) as the secondary antibody (Sigma). HIV-1 Gag proteins were detected with a anti-CA (Serotec) used at 1∶4000 dilution and a HRP anti-goat (1∶2000) as the secondary antibody (Sigma). Actin proteins were detected with a commercial anti-actin antibody (Sigma) used at a 1∶500 dilution and a HRP anti-rabbit (1∶2000) as secondary antibody. Fluorescence was recorded by a CCD chemiluminescence camera system (Gnome, Syngene) and quantified by ImageQuant software.

## Results

### Role of the NC basic sequence and zinc finger in virus assembly

**Figure 2:**
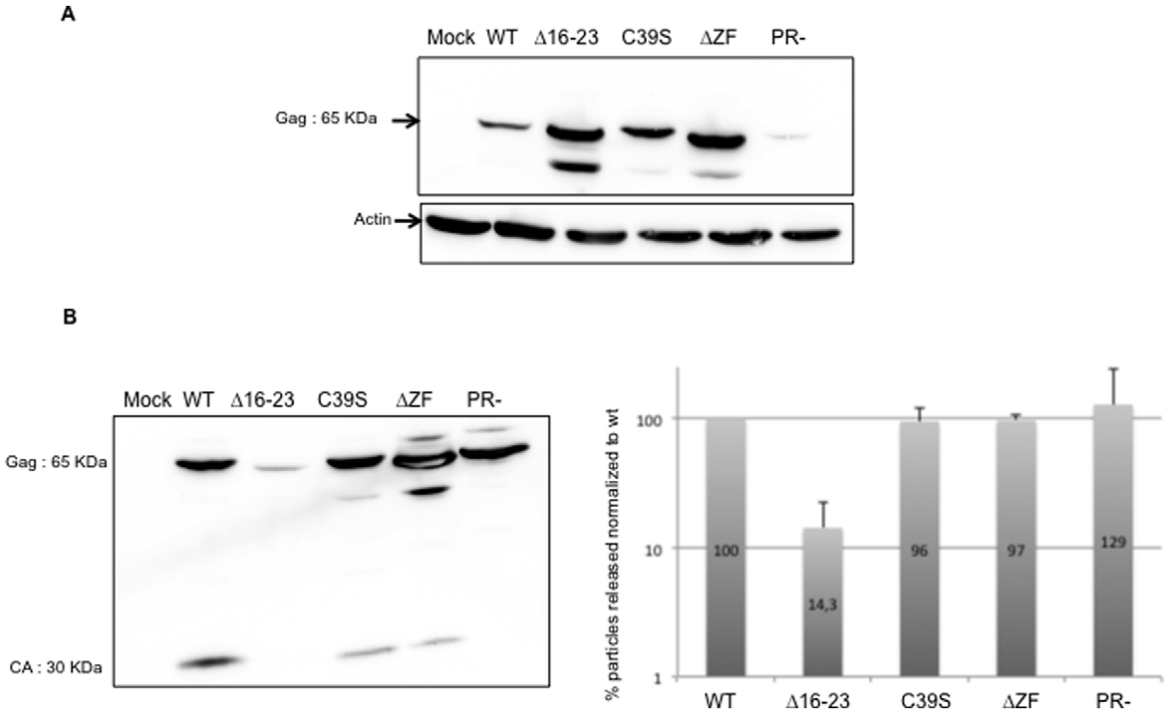
Viral particles produced by MuLV producer cells. (A) MuLV expression was analysed in cells by immunoblotting with an anti-CA antibody. Actin was probed as a loading control. (B) Mature capsid (CA) and Gag were detected in viral samples. Signals were quantified with ImageQuant software. For each lane, signals corresponding to all the bands were added and normalized to wt level (right part). Error bars indicate SD from at least three independent experiments.

The conserved basic residues and the unique ZF of MuLV NC are important functional determinants in virus replication (Fig 1). To study their role in MuLV assembly, we used full-length molecular clones with mutation or deletion in the NC domain of Gag (called NC here) (Fig 1). The N-terminal basic residues of NC (Fig 1) were deleted generating the NC Δ16–23 mutant clone lacking residues R16 through R23. The ZF motif was either mutated by changing Cys-39 to a Ser (C39S), or completely deleted (ΔZF). In parallel, we used a molecular clone (PR-) where the protease was inactivated by a deletion (nt 2421–2546). Mutating the protease enzyme prevented processing of the two Gag and GagPol precursors. Importantly, inactivating the HIV-1 protease has an impact on intravirion DNA level [29]. Each MuLV molecular clone was transfected into 293T cells and levels of Gag proteins in pelletable viral particles were monitored by immunoblotting, as previously reported [37]. To detect Gag and mature capsid (CA) in cellular and viral samples, immunoblotting was conducted with an anti-CA primary antibody (Fig 2). When virus release was efficient (wt and PR-), Gag did not accumulate in cells. As shown in Fig 2B, the levels of Gag processing varied somewhat, as illustrated by the ratios of CA to Gag proteins. To determine the level of virus produced, signals were quantified with ImageQuant software, normalized to wt level and average values from three independent experiments are given in Fig 2B. Results indicate that the ZF mutants, C39S and ΔZF, produced wt level of viral particles in the culture medium, but these mutant particles contained incompletely processed Gag. This partial Gag processing might explain, at least in part, the loss of MuLV infectivity when mutating the NC cysteines in the zinc finger [8], [38]. As expected, the PR- mutant produced immature virions at wt level. In contrast, deleting the N-ter basic residues (Δ16–23) induced a severe decrease (86%) of MuLV production (Fig 2). The deletion of the basic residues caused a dramatic release defect, while ZF mutation or deletion induced only a default in Gag processing.

### Quantitative analysis of the impact of NC mutations on genomic RNA packaging into virions

NC is thought to drive the interaction of Gag with nucleic acids and as such drives the specific incorporation of the gRNA into assembling viral particles [12] by binding to the 5′ UTR of the gRNA with high affinity (for review [16], [39]). Subsequently, Gag-gRNA complexes reach the plasma membrane where formation of viral particles is completed (for review [18]). As for other retroviral NC's [40], [41] the NC packaging function primarily relies on its ability to interact with nucleic acid sequences, notably the 5′ UTR of the gRNA in a very tight mode, which drives gRNA selection. At the same time NC binding to the gRNA causes genome dimerization chaperoned by the NC annealing activity [42].

Recently, we reported that mutating the NC ZF of HIV-1 resulted in virions where the newly made viral DNA replaced the gRNA, due to the RTion of the gRNA before virus release. This study also showed a correlation between intravirion levels of viral DNA and gRNA among the HIV-1 NC-mutant particles [43]. To determine whether this property was conserved in gammaretroviruses such as MuLV, we first examined the impact of NC mutations on the level of gRNA packaging in a quantitative manner by RT-qPCR.

**Figure 3:**
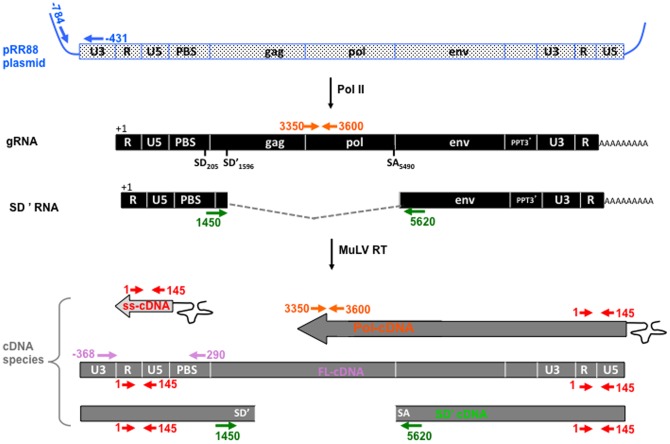
Strategy of qPCR to monitor the MuLV nucleic acid species. Templates and primers used for qPCR analyses were schematically represented. Only the spliced SD' RNA (SD'/SA) important for this study and the gRNA are indicated. A color code was used to illustrate the specificity of the PCR-primer pairs (arrows) that were used to quantify the pR88 plasmid (blue) which generates the MuLV gRNA transcript (orange) and the spliced SD' RNA (green). Numbers refer to the position of the elongation start. Bottom panel: Products of viral reverse transcription. The primer pairs used to detect the intermediate ss-cDNA (red), Pol cDNA (orange), SD' cDNA (green) and the final product FL DNA (purple) are shown.

**Figure 4:**
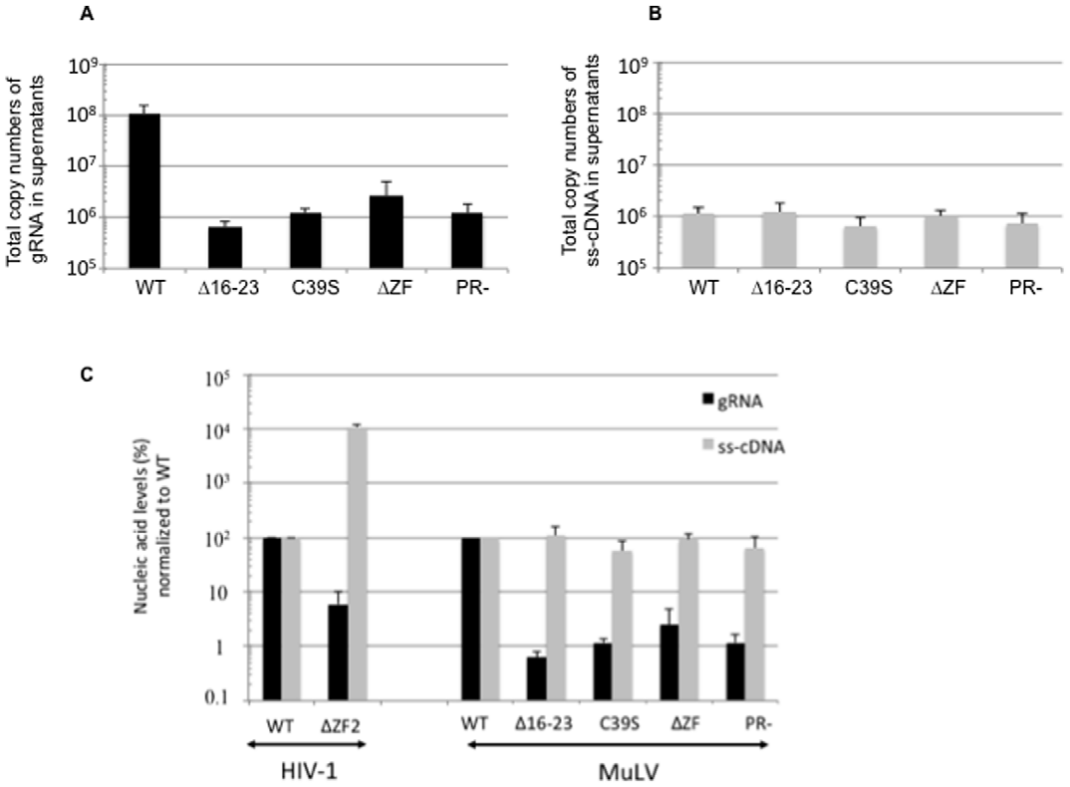
Quantitative analysis of the nucleic acid content of viral particles released from MuLV producer cells. (A) Quantitation of viral gRNA incorporated in wt or mutant viruses by RT-QPCR. Mock controls were subtracted from assays. Error bars indicate SD from at least four independent experiments. (B) Viral DNA levels were determined by qPCR in the wt and mutant virions. DNA was extracted from same virion samples as those used before for gRNA quantitation. Error bars indicate SD from at least seven independent experiments. (C) There is no correlation between gRNA and viral DNA levels among the MuLV mutants. For comparative purpose, data obtained with HIV-1 virions deleted of the second ZF (ΔZF2) are given (left part) [26], [38]. To facilitate the comparison, levels of viral gRNA and ss-cDNA were normalized to those measured in wt virions.

For the first time, the ability of MuLV NC to package the gRNA was monitored by RT-qPCR. Identical volumes of MuLV containing medium were collected and MuLV particles pelleted by centrifugation through a sucrose cushion. Next, MuLV samples were treated by RNAse-free DNase before particle lysis to remove any transfected plasmid DNA, which could interfere with the qPCR assays. As an internal control, we used aliquots of NC-mutant HIV-1 virions that contain a high level of viral DNA. This allowed us to monitor the level of the MuLV particle recovery after ultracentrifugation and DNase treatment. Nucleic acids were purified by two successive phenol-chloroform treatments. The recovered RNAs were reverse transcribed using an oligodT primer and quantitative analyses were carried out using PCR primer pairs that specifically target the intronic region of the viral unspliced RNA (Fig 3, top-part). Two controls for the RT-qPCR reactions were systematically included, (i) one to assess DNA contamination by means of a RTion reaction in the absence of any added RT followed by quantitative PCR amplification and (ii) another one to monitor background amplification levels by real-time PCR with a RNA sample purified from mock-transfected cells. The levels of gRNA in virions were determined as copy numbers in virion pellets. Average values are given in Fig 4A and are from 4 independent experiments. As expected, wt virions contain the highest level of gRNA with 10^8^ copies in total culture medium. The MuLV PR- particles contained 80-fold less gRNA than wt MuLV, while cells transfected with the MLV PR- DNA produced a wt level of pelletable Gag in the medium. The MuLV C39S and ΔZF mutants also showed a severe decrease in gRNA incorporation, namely 80 and 40 fold less than in MuLV wt virions, respectively (Fig 4A). As for MuLV PR-, such a decrease was not due to a lower level of Gag in the medium. The MuLV Δ16–23 mutant particles had a drop of 2-orders of magnitude of its gRNA content (160-fold decrease). Such a dramatic decrease was partly caused by a 7-fold decrease of Gag-associated particles combined to a drastic default in gRNA packaging.

In conclusion, all the MuLV NC mutants examined here had a defect in gRNA packaging, at a degree similar to that of the MuLV PR- mutant. These results confirm the critical role of the NC basic residues and ZF on MLV gRNA packaging.

### NC mutations do not result in a high level of viral DNA in MLV virions

We next asked whether NC mutation or deletion of the ZF could promote late RTion resulting in the synthesis of viral DNA and the production of DNA-containing MuLV as previously observed with HIV-1 NC mutants [25], [26]. HIV-1 experiments conducted with a defective protease (PR-) provide evidences supporting that proteolytic processing may cause, at least in part, the late RTion process [29]. This prompted us to examine the DNA content of immature MuLV virions produced by the PR- mutant.

In order to monitor the level of recovery of intravirion MuLV DNA after DNase treatment of the pelleted virions, a calibrated amount of ΔZF2 HIV-1 was added to MuLV supernatant and not to the HIV-1 assays (see methods). The ΔZF2 HIV-1 particles contained 100-fold more viral DNA than wt HIV-1 particles, resulting from an optimal late RTion activity (Fig 4C left part) [26]. For each MuLV assay, a systematic q-PCR was performed to monitor HIV-1 multispliced cDNA added as a tracer, as previously described [26]. To perform an in-depth analysis of the DNA content of the mutant MuLV particles, we used q-PCR which is a sensitive quantitative approach to monitor the levels of the minus strong-stop DNA (ss-DNA), Pol and FL cDNA forms (Fig 3). In parallel, q-PCR amplifications were run with primer pairs specific for the transfected plasmid (pRR88) but not for the newly made viral FL cDNA (Fig 3). The FL and Pol cDNA forms gave copy numbers similar to that obtained with the control pRR88 primers (5±5×10^2^ copies), indicating the absence of detectable copies of FL and Pol DNAs in the wt and mutant MuLV particles. In order to improve sensitivity, we used PCR primer pairs specific for the R-U5 region (named ss-cDNA primers) to sum up all RTion products (Fig 3). Indeed, the R-U5 region is included in the shortest reverse transcripts such as the ss-cDNA in addition to all the intermediate (Pol-DNA) and the full length (FL) cDNAs. Also there is a duplication in the FL DNA at the 5′ and 3′ ends (Fig 3). The pRR88 copy numbers (representing 10% of the ss-cDNA cps) were substracted from the ss-cDNA values and the results from at least 7 independent experiments showed no variation of the ss-cDNA levels among the mutants (Fig 4B). In conclusion there is no variation of the DNA content between the virions with a mutated NC, a defective PR (PR-) and the wt MuLV particles (Fig 4B). Previously, we showed that the alternatively spliced SD' RNA, generated by usage of the SD' and SA splicing sites (Fig 3), is specifically incorporated in wt MuLV and is reverse transcribed as efficiently as the unspliced gRNA [44]. Thus, the spliced SD'-cDNA would be a useful alternative for specific viral DNA quantitation without the requirement to remove the contaminant pRR88 cps. It was not possible to detect specific SD' cDNA forms in neither wt nor NC-mutant viruses. Average levels of spliced SD' cDNA measured in the DNA samples (3×10^2^ copies) were not significantly different from the background level measured with the mock control using culture supernatant collected from mock-transfected cells (see methods). The presence of MuLV cDNA (ss-cDNA and SD' cDNA) in producer cells was examined as previously described [26]. In contrast to HIV-1, the viral cDNA was not found in cellular DNA samples (data not shown). These results are consistent with those on the virion viral DNA content and indicate the absence of active late RTion in MuLV producer cells (Fig 4B).

### Analysis of the coexpression of MuLV and HIV-1

**Figure 5:**
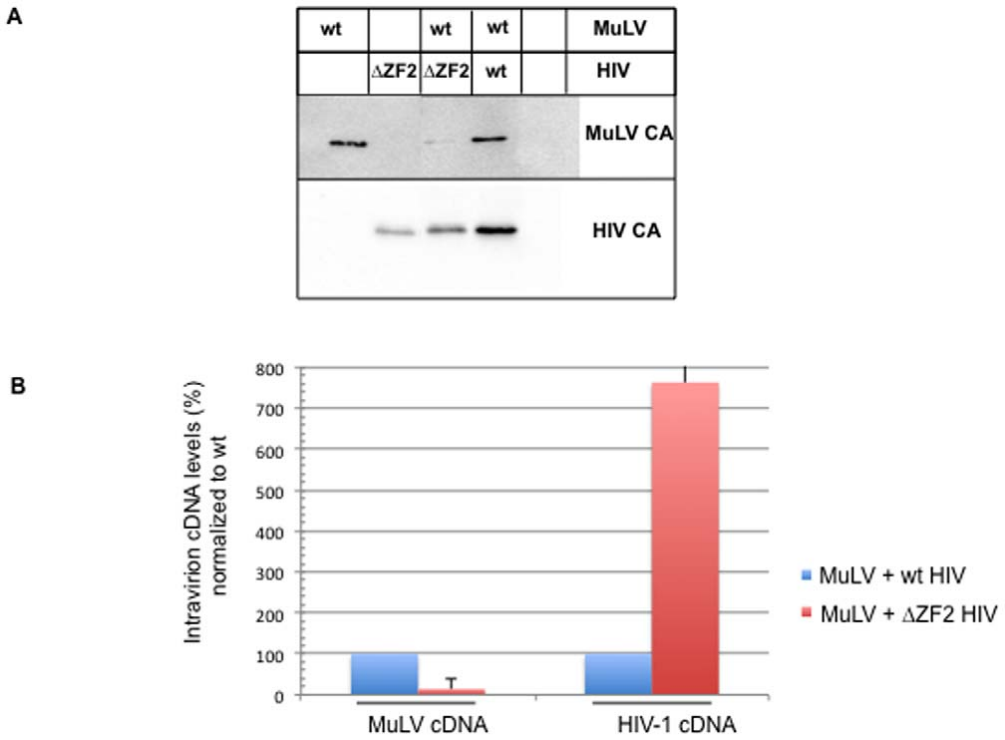
Coproduction of MuLV and HIV-1 virions. Supernatant were collected from cells cotransfected with MuLV and wt or ΔZF2 HIV-1 molecular clones (MuLV:HIV ratio of 1∶3). Released virions were pelleted and proteins analyzed by Western blotting (A). The same blot was used to probe the MuLV and HIV-1 CA proteins. The intravirion levels of MuLV and HIV-1 DNA were determined and calculated as in Fig 4C (B).

MuLV and HIV-1 NCs have similar functions in assembly which is further highlighted by the production of chimeric MuLV-HIV-1 VLPs. In addition, the HIV NC can recognize the MuLV RNA genome although less efficiently than the HIV-1 gRNA [12], [45]. Based on these observations, we wanted to examine whether in the context of complete viruses, the expression of the ΔZF2 HIV-1 mutant, which produced DNA-containing particles, could confer late RTion activity to MuLV. HIV-1 ΔZF2 NC could recognize the MuLV gRNA, causing its reverse transcription as for the HIV-1 gRNA. To this end, MuLV was cotransfected with the wt or ΔZF2 HIV-1 molecular clone (pNL4-3). No HIV tracer was added to the supernatants during these assays. First, we examined the particles released by Western immunoblotting with anti-CA antibodies specific for MuLV or HIV-1 (Fig 5A). Surprisingly, MuLV production was impaired in presence of the ΔZF2 HIV-1 mutant, but not by the wt HIV-1. In contrast, ΔZF2 HIV-1 production remained unchanged with or without MuLV. Then, we analyzed the DNA content of the released particles. Examination of MuLV DNA in virion released when MuLV and ΔZF2 were coexpressed, showed a reduction of the intravirion DNA (Fig 5B). This result correlates with the failure to release virions (Fig 5A). Upon MuLV expression, the level of HIV-1 intravirion DNA was higher in presence of ΔZF2 HIV-1 than with wt HIV-1 (7.5-fold). However, in previous experiments with HIV-1 alone (Fig 4C) the maximum differences observed between the ΔZF2 and wt HIV-1 were about 100-fold. These results suggest that the presence of MuLV impaired the late RTion activity of the mutant HIV-1 (ΔZF2).

## Discussion

NC is involved in the RTion reation with at least two key partners, the RT enzyme and the genomic RNA template (gRNA). In fact, NC molecules extensively coat the gRNA to form the nucleocapsid structure (Darlix et al., 1995; 2011) where tight interactions take place between NC molecules, the cellular tRNA primer and the RT enzyme [2]. The role of NC in RTion largely relies on its nucleic acid chaperone activity, i.e. the ability to direct nucleic acid conformational rearrangements [40], [46]. Moreover, NC exerts a control over the timing of RTion, in a spatio-temporal manner. Indeed, mutating the N-terminal basic residues or the zinc finger motifs (ZF) of HIV-1 NC caused late RTion in HIV-1 producer cells with a 10–100 fold enhancement of newly made viral DNA found in virions as compared with wild-type virions [25], [26], [43]. How HIV-1 controls this late RTion activity remains a matter of debate. However, inactivating the HIV-1 protease or slowing down virus release modulates intravirion DNA levels in such HIV-1 mutants [29], indicating that these two late viral steps are impacting on the timing of RTion.

Structural features of NC tend to be conserved among retroviruses [47]. However, unlike most retroviruses that harbor two ZF motifs, the gammaretroviruses such as MuLV have only one ZF. This feature also distinguishes spumaretroviruses, DNA-containing viruses, which have no NC ZF motif. Also the primary structure of MuLV NC is different from that of HIV-1 since it is more basic. Such MuLV NC unique features prompted us to examine MuLV NC activities by mutating the N-terminal basic residues and the unique ZF motif and monitoring their impact on the late events of MuLV replication. The present study showed that MuLV basic residues are an essential component for virus assembly and gRNA packaging (Fig 2 and 4) and that MuLV (Fig. 4) and HIV-1 [43] ZFs appear to play equivalent role in gRNA packaging. Moreover, we recently reported that mutating basic residues or the ZF of HIV-1 NC resulted in virions containing large amounts of newly made viral DNA, which was generated by RTion of the gRNA before virus release (late RTion) [43]. Such correlation between gRNA and DNA levels was investigated in MuLV NC mutants. We found major differences between MuLV and HIV-1 NC for the temporal control of RTion during virus assembly. Unlike HIV-1, mutations of NC's basic residues or ZF did not turn MuLV into a DNA-containing virus. Only short ss-cDNA forms were found in MuLV particles but not in MuLV producer cells, while intermediate or full-length RTion products remained undetectable (Fig 4C). The viral ss-cDNA synthesis was likely initiated after virus release. It is known that RTion can initiate in newly made viruses. Such natural endogenous RTion activity (NERT) activity produces mainly ss-cDNA, probably because retroviral particles contain insufficient levels of deoxynucleotide triphosphates to complete synthesis of long cDNA products [23], [48], [49]. Moreover, our experiments with MuLV and HIV-1 coexpression (Fig 5) showed for the first time that the ΔZF2 HIV mutant negatively interfered with MuLV assembly or release, but could not promote late RTion in MuLV. Interestingly, MuLV NC restricted the late RTion activity of the ΔZF2 HIV mutant. Consequently, MuLV NC seems to modulate late RTion during assembly of MuLV and HIV-1. Altogether, these results imply that the late RTion and the virus assembly are two linked events.

Why late RTion can take place during assembly of HIV-1 NC mutants but not in the case of MuLV NC mutants? Yet it is not known whether HIV-1 NC directly or indirectly controls the timing of late RTion. As a simple gammaretrovirus, MuLV might miss a cofactor essential for the temporal control of RTion during assembly. In addition, MuLV and HIV-1 NC proteins exhibit differences in their overall chaperone activities in vitro, with a higher activity for HIV-1 NC compared to MuLV NC [42], [50]. Furthermore, HIV-1 NC can directly interact with the RT enzyme promoting RTion processivity [51], [52]. Such NC/RT interactions have never been reported for MuLV replicative nucleoprotein complexes. One explanation could also rely on the gRNA capacities to adopt particular conformation that regulates viral functions. For instance HIV-1 gRNA forms U5:AUG interaction that promotes NC binding and RNA packaging [53]. Such long-distance base-pairing was not reported in the MuLV gRNA [16].

Another explanation might rely on differences in the assembly of MuLV and HIV-1 Gag proteins. Assembly is a well-orchestred process involving three domains of Gag: i) the membrane-binding domain (M) located at the N terminus, ii) The Gag-Gag interaction domain (I) located in the NC sequence and iii) the late (L) domain needed for virus budding and release (for review [17]). The NC basic residues are important for Gag assembly with a possible role in the timing and location of the initial Gag multimerization reaction Comparative studies on HIV-1 and MuLV Gag assembly indicate that MuLV Gag molecules start to interact at much later time after synthesis than those of HIV-1 [54] and with a much weaker protein-protein interaction [55]. A recent study reported that perturbation of the NC N-terminal region caused the assembly of aberrant non-infectious HIV-1 particles but directed the efficient assembly of MuLV particles [56]. This different assembly requirement distinguishes MuLV from other retroviruses and thus timing, Gag trafficking and the rate of virus assembly can possibly impact on the control of RTion during the late phase of virus replication.
